# Enhancing deep chemical reaction prediction with advanced chirality and fragment representation

**DOI:** 10.1039/d5cc02641e

**Published:** 2025-08-26

**Authors:** Fabrizio Mastrolorito, Fulvio Ciriaco, Orazio Nicolotti, Francesca Grisoni

**Affiliations:** a Department of Biomedical Engineering, Institute for Complex Molecular Systems (ICMS) & Eindhoven AI Systems Institute (EAISI), Eindhoven University of Technology Eindhoven The Netherlands f.grisoni@tue.nl; b Dipartimento di Farmacia-Scienze del Farmaco, Università degli Studi di Bari Aldo Moro Bari Italy; c Dipartimento di Chimica, Università degli Studi di Bari Aldo Moro Bari Italy

## Abstract

This work focuses on organic reaction prediction with deep learning, with the recently introduced fragSMILES representation – which encodes molecular substructures and chirality, enabling compact and expressive molecular representation in a textual form. In a systematic comparison with well-established molecular notations – simplified molecular input line entry system (SMILES), self-referencing embedded strings (SELFIES), sequential attachment-based fragment embedding (SAFE) and tree-based SMILES (t-SMILES) – fragSMILES achieved the highest performance across forward- and retro-synthesis prediction, with superior recognition of stereochemical reaction information. Moreover, fragSMILES enhances the capacity to capture stereochemical complexity – a key challenge in synthesis planning. Our results demonstrate that chirality-aware and fragment-level representations can advance current computer-assisted synthesis planning efforts.

## Abstract 2

This work advances computer-assisted synthesis planning *via* chirality-aware and fragment-level molecular string representations.

## Graphical abstract

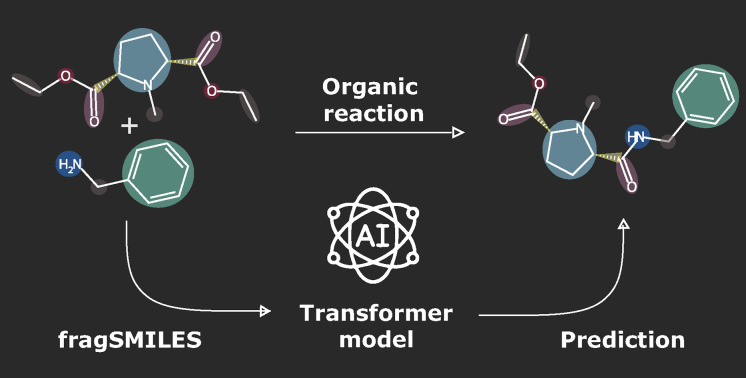

Since time immemorial, operating a chemical laboratory has required patience and meticulous attention to detail, often resulting in long timelines and inconclusive outcomes. In the last decades, artificial intelligence has increasingly supported chemists in expediting their experiments, through machine learning algorithms for process and molecule optimization^1–3^ and robotics-assisted laboratories that streamline the execution.^4,5^ Among these advances, computer-assisted synthesis planning has been particularly transformed by the advent of deep learning,^6,7^ which has demonstrated high accuracy and has significantly reduced the time and resources required compared to traditional trial-and-error approaches.^7–9^

Methods based on string representations of chemicals and organic reactions have gained particular traction,^10^ thanks to their ability to leverage natural language processing techniques.^11,12^ In particular, reactants (or product) molecules are represented as strings, to subsequently predict the product (or reactants) molecules using machine translation models.^9,13^ Popular string notations for synthesis planning^13–17^ include the simplified molecule input line entry system (SMILES^18^) strings, self-referencing embedded strings (SELFIES^19^), sequential attachment-based fragment embedding (SAFE^20^) and tree-based SMILES (t-SMILES^21^).

As chemical reactions involve local molecular changes (leading to a significant overlap of reactants and products), several methods have focused on substructure-based reasoning – for example, extracting preserved molecular fragments to guide decoding,^22^ refining precursor structures through targeted string editing,^23^ or assembling molecules around conserved cores.^24^ Moreover, substructure-based string representations have recently emerged^20,25,26^ to enhance the expressiveness and interpretability of molecular notations, by capturing chemically meaningful fragments and their connectivity. FragSMILES was recently developed for *de novo* molecule design,^26^ to overcome limitations of existing string representations in capturing substructure information, by denoting the fragments independently of the connector atoms, as well as capturing chirality.^27–29^ The fragSMILES algorithm (Fig. 1a) operates by (1) disassembling molecules *via* predefined cleavage rules (exo-cyclic single bonds in this study), (2) collapsing the resulting fragments into the edges of a reduced graph, while keeping track of the atoms connecting the fragments, and (3) converting this graph into a string, whose elements (‘tokens’) represent nodes or edges.

**Fig. 1:**
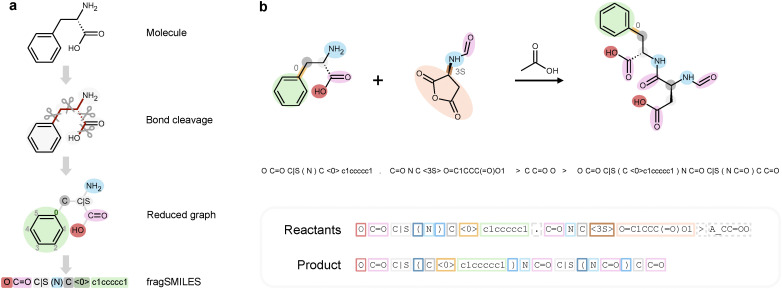
FragSMILES notation for reaction prediction. (a) Molecules are converted into a reduced graph, obtained *via* exocyclic bond cleavage (Sup. Fig. 1). The resulting fragments (nodes) and their connecting bonds (edges) are then converted into elements (‘tokens’) that constitute the fragSMILES string. (b) Exemplary chemical reaction (from reagents to product), encoded as fragSMILES strings. Spaces were added to highlight token separation.

In this study, we apply fragSMILES for synthesis planning, under the hypothesis that its ability to encode substructures and advanced chirality can also enhance reaction prediction and retrosynthesis accuracy. We focused on two tasks: (1) forward reaction prediction, where the goal is to predict the products of a given set of reactants, and (2) retrosynthesis prediction, where the goal is to identify potential reactants and reagents needed to synthesize a target molecule. To this end, we used 1 002 602 curated chemical reactions from the USPTO database^30^ and represented them with different string notations. SMILES, SELFIES, SAFE, and t-SMILES were used as benchmarks. Other notable string representations exist (*e.g.*, DeepSMILES,^31^ GroupSELFIES,^25^ and GenSMILES^32^), which were not considered due to their limited application to organic reaction prediction. SMILES, SELFIES, SAFE and t-SMILES were tokenized at the atom-level. FragSMILES were tokenized at the ‘chemical-word’ level, leading to remarkably more compact sequences^26^ (Sup. Table 1 and Sup. Fig. 3). This characteristic might help mitigate the memory usage associated with the increased complexity of word-level languages.^33,34^ We used the transformer architecture^35^ – the *de facto* standard for organic reaction planning^36^ – and framed the prediction task as a sequence-to-sequence translation (*i.e.*, reactants to reagents, or the other way around) problem.^13,14^ Models were optimized and trained separately for each representation and task (Sup. Tables 2 and 3), and used to generate molecular strings *via* beam search^37^ (see SI). The transformer models were evaluated on 50 234 reactions (unseen during model optimization or training) by measuring (Table 1) (a) validity, *i.e.*, the number of ‘chemically-valid’ strings generated, including correct stereocenter assignations, and (b) accuracy, computed as the number of correct predictions over the total of considered predictions (from top-1 to top-5 sequences).

**Table 1:** Prediction accuracy of SMILES, SELFIES, SAFE, t-SMILES and fragSMILES, on the total set of reactions considered and on a subset of reactions involving stereocenters. Results are reported for both reaction prediction and for retrosynthesis prediction, in terms of validity (*i.e.*, number of ‘chemically valid’ strings generated) and of top-*k* accuracy (50 234 in total, and 8588 when considering reactions involving stereocenters). Metrics are analysed for the top-*k* generations (from 1 to 5) of beam search. Best (bold) and the second best (underline) metrics are highlighted

Task	Metric	Notation	Top-1	Top-2	Top-3	Top-4	Top-5
Forward synthesis	Validity^a^	SMILES	48 366 (96.3%)	49 470 (98.5%)	49 798 (99.1%)	4̲9̲ _9̲2̲7̲ (9̲9̲._4̲%)	5̲0̲ _0̲0̲5 (9̲9̲._5̲%)
		SELFIES	48 418 (96.4%)	48 857 (97.3%)	49 075 (97.7%)	49 213 (98.0%)	49 325 (98.2%)
		SAFE	46 619 (92.8%)	48 020 (95.6%)	48 544 (96.6%)	48 824 (97.2%)	49 008 (97.6%)
		t-SMILES	**50 231 (100.0%)**	**50 234 (100.0%)**	**50 234 (100.0%)**	**50 234 (100.0%)**	**50 234 (100.0%)**
		fragSMILES	4̲8̲ _8̲7̲9 (9̲7̲._3̲%)	4̲9̲ _5̲5̲3 (9̲8̲._6̲%)	4̲9̲ _8̲1̲2 (9̲9̲._2̲%)	49 918 (99.4%)	49 989 (99.5%)
	Accuracy	SMILES	2̲5̲ _0̲5̲3 (4̲9̲._9̲%)	2̲9̲ _2̲6̲1̲ (5̲8̲._2̲%)	3̲1̲ _1̲3̲3̲ (6̲2̲._0̲%)	3̲2̲ _3̲0̲5̲ (6̲4̲._3̲%)	3̲2̲ _9̲8̲8̲ (6̲5̲._7̲%)
		SELFIES	10 538 (21.0%)	13 415 (26.7%)	14 911 (29.7%)	15 904 (31.7%)	16 591 (33.0%)
		SAFE	15 151 (30.2%)	18 758 (37.3%)	20 557 (40.9%)	21 609 (43.0%)	22 169 (44.1%)
		t-SMILES	3087 (6.1%)	4358 (8.7%)	5125 (10.2%)	5611 (11.2%)	6013 (12.0%)
		fragSMILES	**26 826 (53.4%)**	**30 287 (60.3%)**	**32 026 (63.8%)**	**33 015 (65.7%)**	**33 692 (67.1%)**
Retro-synthesis	Validity^a^	SMILES	20 924 (41.7%)	28 481 (56.7%)	33 894 (67.5%)	37 763 (75.2%)	40 743 (81.1%)
		SELFIES	**40 042 (79.7%)**	**45 139 (89.9%)**	**47 366 (94.3%)**	**48 397 (96.3%)**	**48 968 (97.5%)**
		SAFE	21 890 (43.6%)	28 193 (56.1%)	32 939 (65.6%)	36 289 (72.2%)	39 018 (77.7%)
		t-SMILES	3̲6̲ _8̲0̲5̲ (7̲3̲._3̲%)	4̲1̲ _1̲8̲8̲ (8̲2̲._0̲%)	4̲4̲ _2̲2̲8̲ (8̲8̲._0̲%)	4̲5̲ _9̲3̲2̲ (9̲1̲._4̲%)	4̲7̲ _0̲4̲7̲ (9̲3̲._7̲%)
		fragSMILES	28 054 (55.8%)	35 323 (70.3%)	39 682 (79.0%)	42 443 (84.5%)	44 369 (88.3%)
	Accuracy	SMILES	4̲0̲3̲1̲ (8̲._0̲%)	5̲6̲0̲2̲ (1̲1̲._2̲%)	6̲7̲0̲9̲ (1̲3̲._4̲%)	7̲5̲9̲0̲ (1̲5̲._1̲%)	8̲3̲0̲2̲ (1̲6̲._5̲%)
		SELFIES	8 (0.0%)	19 (0.0%)	29 (0.1%)	36 (0.1%)	49 (0.1%)
		SAFE	3731 (7.4%)	4886 (9.7%)	5674 (11.3%)	6392 (12.7%)	6978 (13.9%)
		t-SMILES	0 (0.0%)	0 (0.0%)	0 (0.0%)	0 (0.0%)	0 (0.0%)
		fragSMILES	**4230 (8.4%)**	**6129 (12.2%)**	**7588 (15.1%)**	**8905 (17.7%)**	**10** **091 (20.1%)**
Forward synthesis (chiral)	Validity^a^	SMILES	8088 (94.2%)	8298 (96.6%)	8404 (97.9%)	8444 (98.3%)	8480 (98.7%)
		SELFIES	6847 (79.7%)	7267 (84.6%)	7478 (87.1%)	7609 (88.6%)	7712 (89.8%)
		SAFE	7814 (91.0%)	8026 (93.5%)	8099 (94.3%)	8142 (94.8%)	8182 (95.3%)
		t-SMILES	**8587 (100.0%)**	**8588 (100.0%)**	**8588 (100.0%)**	**8588 (100.0%)**	**8588 (100.0%)**
		fragSMILES	8̲2̲3̲9̲ (9̲5̲._9̲%)	8̲3̲8̲4̲ (9̲7̲._6̲%)	8̲4̲4̲9̲ (9̲8̲._4̲%)	8̲4̲8̲0̲ (9̲8̲._7̲%)	8̲4̲9̲8̲ (9̲9̲._0̲%)
	Accuracy	SMILES	3̲3̲3̲1̲ (3̲8̲._8̲%)	4̲1̲4̲4̲ (4̲8̲._3̲%)	4̲4̲7̲6̲ (5̲2̲._1̲%)	4̲6̲7̲8̲ (5̲4̲._5̲%)	4̲8̲0̲9̲ (5̲6̲._0̲%)
		SELFIES	1170 (13.6%)	1548 (18.0%)	1732 (20.2%)	1859 (21.6%)	1956 (22.8%)
		SAFE	1609 (18.7%)	2095 (24.4%)	2343 (27.3%)	2495 (29.1%)	2575 (30.0%)
		t-SMILES	80 (0.9%)	126 (1.5%)	162 (1.9%)	177 (2.1%)	193 (2.2%)
		fragSMILES	**3801 (44.3%)**	**4345 (50.6%)**	**4652 (54.2%)**	**4825 (56.2%)**	**4957 (57.7%)**
Retro-synthesis (chiral)	Validity^a^	SMILES	3425 (39.9%)	4576 (53.3%)	5551 (64.6%)	6255 (72.8%)	6760 (78.7%)
		SELFIES	**6421 (74.8%)**	**7356 (85.7%)**	**7816 (91.0%)**	**8029 (93.5%)**	**8142 (94.8%)**
		SAFE	3823 (44.5%)	4793 (55.8%)	5563 (64.8%)	6082 (70.8%)	6524 (76.0%)
		t-SMILES	6̲1̲6̲7̲ (7̲1̲._8̲%)	6̲8̲1̲7̲ (7̲9̲._4̲%)	7̲3̲1̲6̲ (8̲5̲._2̲%)	7̲5̲9̲7̲ (8̲8̲._5̲%)	7̲8̲0̲1̲ (9̲0̲._8̲%)
		fragSMILES	4485 (52.2%)	5678 (66.1%)	6452 (75.1%)	6958 (81.0%)	7318 (85.2%)
	Accuracy	SMILES	**669 (7.8%)**	**933 (10.9%)**	1̲1̲0̲8̲ (1̲2̲._9̲%)	1̲2̲4̲9̲ (1̲4̲._5̲%)	1̲3̲4̲3̲ (1̲5̲._6̲%)
		SELFIES	8 (0.1%)	19 (0.2%)	27 (0.3%)	32 (0.4%)	43 (0.5%)
		SAFE	6̲3̲5̲ (7̲._4̲%)	805 (9.4%)	924 (10.8%)	1048 (12.2%)	1125 (13.1%)
		t-SMILES	0 (0.0%)	0 (0.0%)	0 (0.0%)	0 (0.0%)	0 (0.0%)
		fragSMILES	620 (7.2%)	9̲1̲9̲ (1̲0̲._7̲%)	**1128 (13.1%)**	**1297 (15.1%)**	**1469 (17.1%)**

a Computed by considering both syntactic validity (Sup. Table 4) and correct chirality annotation.

t-SMILES consistently achieved 100% validity on forward synthesis prediction with fragSMILES achieving the second highest validity in the top-three generated candidates (Table 1). On retrosynthesis prediction, SELFIES achieved the highest validity (74.8%), with t-SMILES consistently achieving the second highest validity (73.3%). In terms of accuracy, fragSMILES always yielded the highest accuracy in both forward- and retro-synthesis prediction, with at least 204 to 1784 more correct predictions in the top-1. SMILES strings resulted in the second-best performance.

When analysing the substructure similarity between wrong predictions and the correct outcome (forward synthesis, Tanimoto coefficient on extended connectivity fingerprints^38^), all models exhibited comparable trends, with SELFIES and t-SMILES consistently showing lower similarity values on average (Sup. Fig. 4). Additionally, only limited overlap of correct predictions was observed among models using different notations (Sup. Fig. 5), suggesting that each representation captures distinct features of the underlying chemistry. The highest overlaps were found between SMILES and fragSMILES, ranging from 66% in top-1 to 78% in top-5 predictions, indicating some redundancy but also a degree of complementarity across models.

Moreover, we analysed the accuracy of fragSMILES on chemical reactions involving at least one stereocenter from the reactants or chemical product (8588 chemical reactions) as annotated in the original dataset (Table 1). For forward synthesis prediction, fragSMILES outperformed all tested methods, especially visible in the top-1 predictions, with differences in accuracy up to +5%. For retrosynthesis prediction, SMILES slightly outperformed fragSMILES in top-1 accuracy (+0.6%). The validity of SELFIES-generated molecules decreases when focusing on chiral compounds, highlighting the challenge of correctly capturing stereochemistry. The accuracy gap between SAFE and SELFIES further supports this observation. The overlap of accurate predictions between models is reported in Sup. Fig. 6. Neither sequence length, sampling probability nor token frequency could alone explain the general accuracy gains of fragSMILES. We analysed different subsets of reactions involving stereocenters to assess the predictive accuracy of fragSMILES. Across most subsets, fragSMILES was the top-performing representation (Sup. Table 5). The exception was stereoselective reactions, where fragSMILES ranked second (Sup. Table 5).

Finally, we examined the causes of invalid syntax (Fig. 2a)^39^ in forward reaction prediction. SELFIES primarily fails due to incorrect chirality assignments, while the fragment-level tokenization of fragSMILES eliminates syntax errors in cyclic structures (assigned to a single token). However, fragSMILES exhibits issues in bond assignment between fragments, as connector tokens dominate its sequences. Due to its atom-based tokenization, the SMILES language is more prone to errors involving ring closures and branches. In terms of inaccurate predictions (Fig. 2b), fragSMILES outperforms the other notations in correctly predicting cyclic substructures and scaffolds, whereas SMILES has an edge in generating acyclic substructures, reflecting the strengths of each respective representation.

**Fig. 2:**
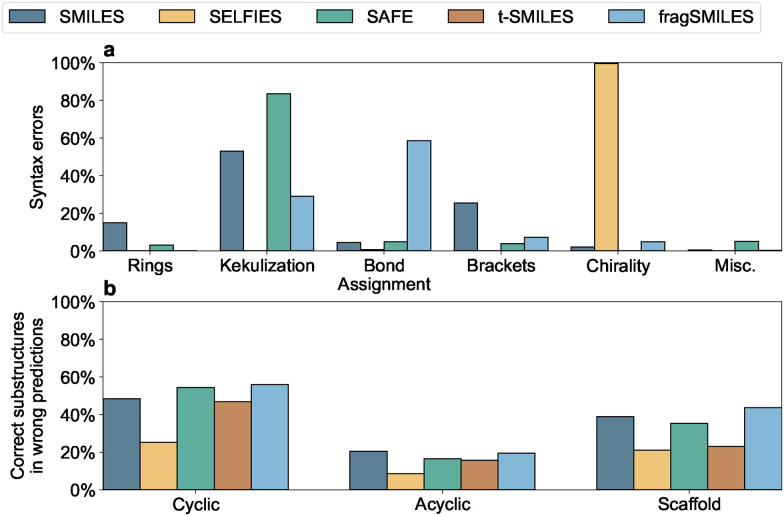
Top-1 predictions (forward synthesis) per representation. (a) Syntax errors of invalid predictions; (b) correctly generated substructures among incorrectly predicted products (grouped by substructure).

This study demonstrates that the fragSMILES language represents an advancement in synthesis planning using deep learning, offering enhanced accuracy and validity over traditional string-based representations like SMILES and SELFIES. By leveraging substructure-based tokenization, fragSMILES captures the complexity of molecular stereocenters and cyclic structures, addressing key limitations in current methods. Its performance, especially in top-1 predictions, underscores its potential for enhancing reaction design and retrosynthetic planning, and becoming one of the *de facto* representations in the field. As AI-driven synthesis tools become more integrated into real-world applications, the ability to predict molecular transformations with high precision is critical, and fragSMILES can contribute to this evolution.

The USPTO dataset, while widely used as a benchmark, has known limitations.^40,41^ Incorporating more rigorous data curation, especially when dealing with stereochemistry, will further benefit the field. Future work integrating fragSMILES with more advanced machine learning techniques (*e.g.*, large language models^42^) or in combination with complementary molecular representations (*e.g.*, molecular graphs), might further push the boundaries of chemical automation.

**Author contributions:** Conceptualization: FM and FG. Data curation: FM and FC. Formal analysis: FM, FG, ON. Investigation: all authors. Methodology: FM and FG. Software: FM. Visualization: FM and FG. Writing – original draft: FM and FG. Writing – review and editing: all authors.

This research was co-funded by the European Union (ERC, ReMINDER, 101077879 to FG). Views and opinions expressed are, however, those of the author(s) only and do not necessarily reflect those of the European Union or the European Research Council.

## Conflicts of interest

There are no conflicts to declare.

## Supplementary Material

CC-061-D5CC02641E-s001

## Data Availability

Supplementary information: Materials and methods, Sup. Fig. 1–6, and Sup. Tables 1–5. See DOI: https://doi.org/10.1039/d5cc02641e All the code and data useful to reproduce the results of this study are available on GitHub at the following URL: https://github.com/molML/fragSMILES4reaction.
