# Supplementary material for: Enhancing deep chemical reaction prediction with advanced chirality and fragment representation
Source: Chem Commun (Camb). 2025 Aug 26;61(93):18344–7. doi: 10.1039/d5cc02641e (PMC12424581; doi:10.1039/d5cc02641e)
Supplement: CC-061-D5CC02641E-s001 [file CC-061-D5CC02641E-s001.pdf]

Supplementary Information (SI) for ChemComm.
This journal is © The Royal Society of Chemistry 2025

Electronic Supporting Information

Enhancing deep chemical reaction prediction with advanced chirality and fragment representation

Fabrizio Mastrolorito, Fulvio Ciriaco, Orazio Nicolotti, and Francesca Grisoni

Contents

Materials and Methods 2
- SMILES tokenization . . . 2
- Data preparation and processing . . . 2
- Model optimization and selection . . . 2
- Molecule Sampling . . . 2
- Software and Code . . . 2

Supporting Figures 3

Supporting Tables 9

Materials and Methods

SMILES tokenization

Chemical reactions represented as SMILES , SAFE and t-SMILES notations were split into encoded unit blocks adopting the atom-based tokenization which respects the following regex pattern:

```
\[[^\]]+]|Br?|Cl?|N|O|S|P|F|I|b|c|n|o|s|p|\(|\)|\.|=|#|-
|\+|\\|\/|:|~|@|\?|>>?|\*|\$|\%[0-9]{2}|[0-9]|&|\^
```

Data preparation and processing

The SMILES strings of the original database[1] were curated by removing geometric stereochemical information. SMILES strings were canonicalized and tokenized at the atom-level (see Supporting Information). SMILES strings were converted into the corresponding fragSMILES, and tokenized at the fragment-level by identifying fragments, brackets, and atom index tags as a unique block to tokenize. Only molecules whose SMILES and fragSMILES sequences had a maximum length of $\leq$ 200 tokens (SMILES) and of $\leq$ 150 tokens (fragSMILES) were retained for model training. In total, 1,002,602 unique reactions were retained for model training, which were randomly split into a training, validation, and a test set of 902,255 (90%), 50,113 (5%), and 50,234 (5%) chemical reactions, respectively. After tokenization, molecular strings were encoded into integer values to serve as model input.

Model optimization and selection

The transformer models[2] all consisted of an input embedding layer, a positional encoding layer, a multi-layer transformer encoder, and a transformer decoder. Token vocabulary was shared among the encoder and the decoder. Hyperparameter tuning was performed on embedding size, number of layers, number of heads per layer, learning rate, and batch size (Sup. Table 2), via grid search, using cross-entropy loss. Early stopping was applied during training when the training loss did not vary by more than `0.02` for two consecutive epochs. The best model for each task and each representation (Sup. Table 3) was selected based on validation loss, *i.e.* the lowest value one.

Molecule Sampling

The sampling phase to predict chemical product or reactants, up to five most probable sequences, was conducted by Beam search algorithm. Specifically, the starter token was employed to initialize the beam and shared among generated sequences. Then, the five most next probable tokens were concatenated to the starter token to obtain five sequences. Probability for each next token is computed as logarithmic softmax function and summed to the previous generation. The most probable five sequences are selected from all possible generations ($5 \cdot 5 = 25$ in this case) of the beams. When the closure token occurs for one sampled sequence, that generation is ranked as the top-i probable generation. When the closure token appears simultaneously for multiple sequences, the ranking is sorted by logit value.

Software and Code

All calculations were performed in a Python (v 3.12.8) environment. Models were optimized and trained using PyTorch (v 2.3.1) and PyTorchLightning (v 2.4.0). Molecule handling was performed with RDKit (v 2024.3.3). SMILES strings were converted into fragSMILES notation by using the algorithm provided at GitHub URL: `https://github.com/f48r1/chemicalgof` where descriptions, documentations and tutorial are included as well. Full Python scripts to replicate this study are available on GitHub repository at the following URL: `https://github.com/molML/fragSMILES4reaction`. A static version of this repository will be published on Zenodo upon paper acceptance.

Supporting Figures

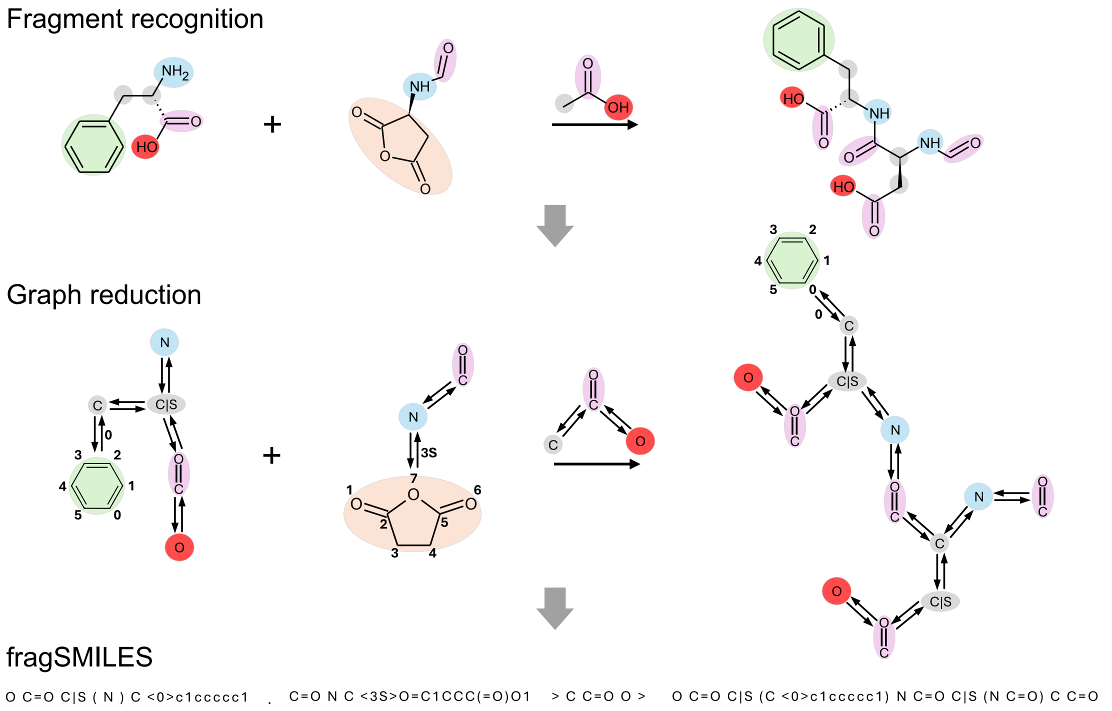

Sup. Fig 1 fragSMILES procedure. The fragment recognition is allowed by a defined fragmentation rule; the reduced graph is composed of fragments as nodes and bidirectional edges to track connector atoms between substructures.

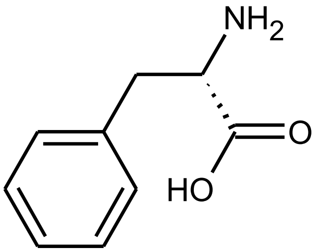

SMILES `N [C@@H] ( C c 1 c c c c c 1 ) C ( = O ) O`

SELFIES `[N][C@@H1][Branch1][#Branch2][C][C][=C][C][=C][C][=C][Ring1][=Branch1][C][=Branch1][C][=O][O]`

SAFE `N [C@@H] ( C 2 ) C ( = O ) O . c 1 2 c c c c c 1`

t-SMILES `* N & * [C@@H] ( * ) * & * C * & * C 1 = C C = C C = C 1 ^ * C ( = O ) O & & & &`

fragSMILES `O C=O C|S ( N ) C <0> c1ccccc1`

Sup. Fig 2 Different chemical representations. A Generic molecular structure represented as SMILES, SELFIES, SAFE and fragSMILES notations.

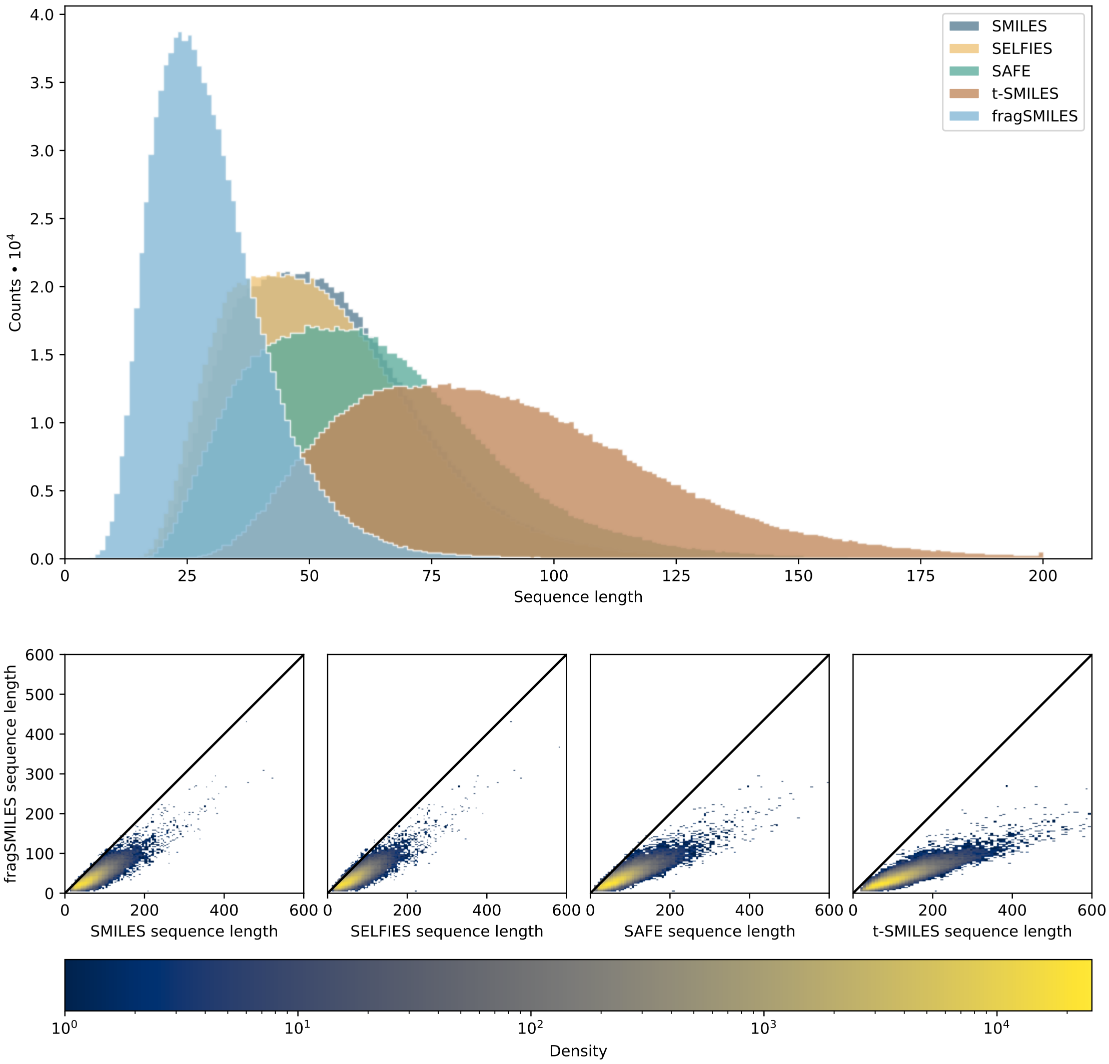

Sup. Fig 3 Lengths of encoded chemical reaction sequences represented as different chemical notationns: upper side) global length values distribution; lower side) density plot of pair-wise correlated lengths between fragSMILES and the other chemical notations.

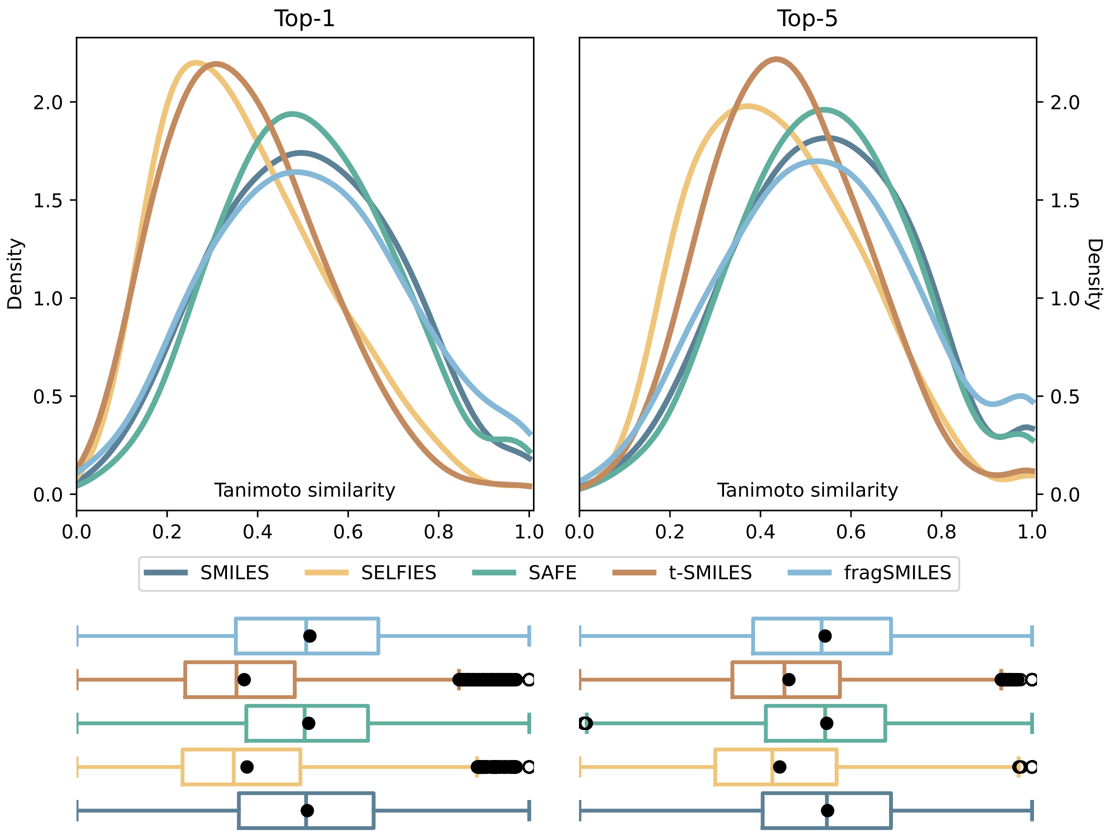

Sup. Fig 4 Similarity values computed between (a) the correct molecules and (b) valid but erroneous molecules predicted by each model. The distribution of molecular similarity as computed on extended connectivity fingerprints (RDKit, default settings) is reported.

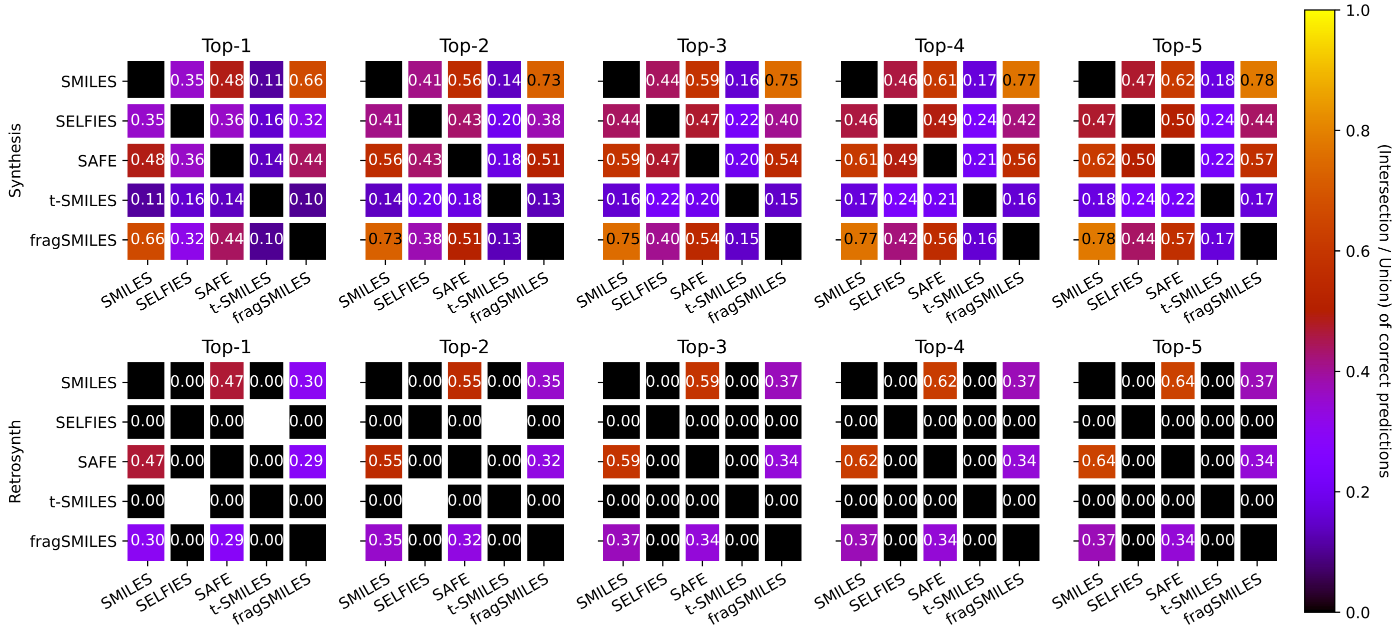

Sup. Fig 5 Heatmap concerning the values of the ratio between the set of the intersection and union of the correctly predicted molecules for each chemical notation pair. The top and bottom rows refer to the predictions of synthesis and retrosynthesis, respectively, from Top-1 (from left) to Top-5 (right) generations.

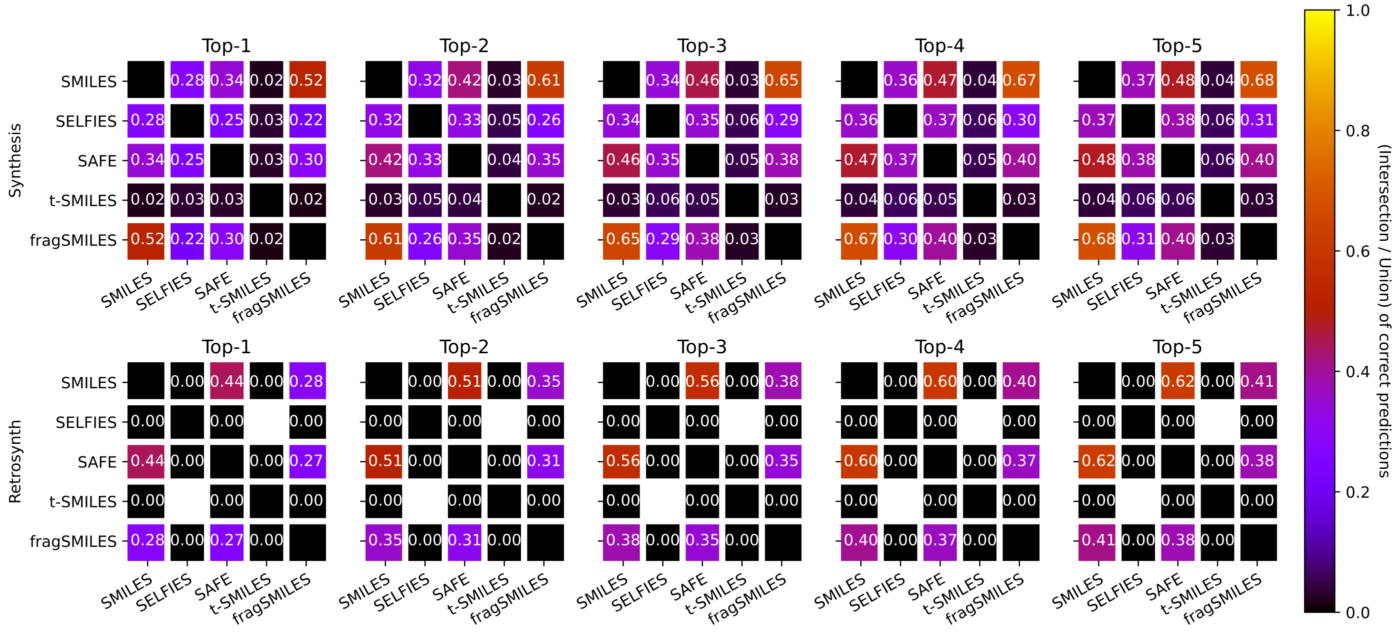

Sup. Fig 6 Heatmap concerning the values of the ratio between the set of the intersection and union of the correctly predicted chiral molecules for each chemical notation pair. The top and bottom rows refer to the predictions of synthesis and retrosynthesis, respectively, from Top-1 (from left) to Top-5 (right) generations.

Supporting Tables

Sup. Table 1 Mean length values returned by each chemical notation on reaction sequences dataset.

Language	Length (mean ± dev. std.)
SMILES	54 ± 20
SELFIES	52 ± 20
SAFE	62 ± 25
t-SMILES	90 ± 34
fragSMILES	29 ± 12

Sup. Table 2 Hyperparameter space explored for each transformer model on each task.

Hyperparameters	model_dim	num_heads	num_layers	batch_size	lr	dropout
Adopted values	(256, 512)	(1, 2, 3, 4)	(1, 2, 3, 4, 5)	(256, 512)	(0.001, 0.0001)	0.3

Sup. Table 3 Hyperparameter values for each representation and task.

model_dim	num_heads	num_layers	batch_size	lr	dropout	notation	task
256	4	4	512	0.001	0.3	SMILES	Synthesis
256	4	3	512	0.001	0.3	SELFIES	Synthesis
512	4	5	256	0.0001	0.3	SAFE	Synthesis
256	4	2	512	0.001	0.3	t-SMILES	Synthesis
512	4	5	256	0.0001	0.3	fragSMILES	Synthesis
256	4	4	512	0.001	0.3	SMILES	Retrosynth.
256	4	3	256	0.001	0.3	SELFIES	Retrosynth.
256	4	4	512	0.001	0.3	SAFE	Retrosynth.
512	4	2	512	0.001	0.3	t-SMILES	Retrosynth
512	4	5	256	0.0001	0.3	fragSMILES	Retrosynth.

Sup. Table 4 Prediction accuracy of SMILES, SELFIES, SAFE and fragSMILES, on the total set of reactions considered and on a subset of reactions involving chirality. Results are reported for both reaction prediction and for retrosynthesis prediction, in terms of validity (i.e., number of 'chemically valid' strings generated) and of top-k accuracy (50,234 in total, and 8588 when considering reactions involving chirality). Metrics are analysed for the top-k generations (from 1 to 5) of beam search. For each metric and generation, the best and the second best performing values are highlighted in boldface and underlined, respectively.

Task	Metric	Notation	Top-1	Top-2	Top-3	Top-4	Top-5
Forward Synthesis	Validity	SMILES	48404 (96.4%)	49495 (98.5%)	49804 (99.1%)	49932 (99.4%)	50009 (99.6%)
		SELFIES	50224 (100.0%)	50227 (100.0%)	50227 (100.0%)	50228 (100.0%)	50229 (100.0%)
		SAFE	46619 (92.8%)	48020 (95.6%)	48544 (96.6%)	48824 (97.2%)	49008 (97.6%)
		t-SMILES	**50231 (100.0%)**	**50234 (100.0%)**	**50234 (100.0%)**	**50234 (100.0%)**	**50234 (100.0%)**
		fragSMILES	48946 (97.4%)	49594 (98.7%)	49834 (99.2%)	49937 (99.4%)	50007 (99.5%)
	Accuracy	SMILES	25053 (49.9%)	29264 (58.3%)	31136 (62.0%)	32308 (64.3%)	32991 (65.7%)
		SELFIES	10538 (21.0%)	13415 (26.7%)	14911 (29.7%)	15904 (31.7%)	16591 (33.0%)
		SAFE	15151 (30.2%)	18758 (37.3%)	20557 (40.9%)	21609 (43.0%)	22169 (44.1%)
		t-SMILES	3087 (6.1%)	4358 (8.7%)	5125 (10.2%)	5611 (11.2%)	6013 (12.0%)
		fragSMILES	**26830 (53.4%)**	**30291 (60.3%)**	**32030 (63.8%)**	**33020 (65.7%)**	**33697 (67.1%)**
Retro-synthesis	Validity	SMILES	20926 (41.7%)	28486 (56.7%)	33904 (67.5%)	37771 (75.2%)	40754 (81.1%)
		SELFIES	**40541 (80.7%)**	**45449 (90.5%)**	**47552 (94.7%)**	**48526 (96.6%)**	**49066 (97.7%)**
		SAFE	21890 (43.6%)	28193 (56.1%)	32939 (65.6%)	36289 (72.2%)	39018 (77.7%)
		t-SMILES	36805 (73.3%)	41188 (82.0%)	44228 (88.0%)	45932 (91.4%)	47047 (93.7%)
		fragSMILES	28054 (55.8%)	35323 (70.3%)	39682 (79.0%)	42443 (84.5%)	44369 (88.3%)
	Accuracy	SMILES	4018 (8.0%)	5587 (11.1%)	6695 (13.3%)	7577 (15.1%)	8290 (16.5%)
		SELFIES	0 (0.0%)	0 (0.0%)	2 (0.0%)	3 (0.0%)	3 (0.0%)
		SAFE	3731 (7.4%)	4886 (9.7%)	5674 (11.3%)	6392 (12.7%)	6978 (13.9%)
		t-SMILES	0 (0.0%)	0 (0.0%)	0 (0.0%)	0 (0.0%)	0 (0.0%)
		fragSMILES	**4230 (8.4%)**	**6128 (12.2%)**	**7587 (15.1%)**	**8904 (17.7%)**	**10089 (20.1%)**
Forward Synthesis (chiral)	Validity	SMILES	8126 (94.6%)	8323 (96.9%)	8410 (97.9%)	8449 (98.4%)	8484 (98.8%)
		SELFIES	**8588 (100.0%)**	**8588 (100.0%)**	**8588 (100.0%)**	**8588 (100.0%)**	**8588 (100.0%)**
		SAFE	7814 (91.0%)	8026 (93.5%)	8099 (94.3%)	8142 (94.8%)	8182 (95.3%)
		t-SMILES	8587 (100.0%)	8588 (100.0%)	8588 (100.0%)	8588 (100.0%)	8588 (100.0%)
		fragSMILES	8296 (96.6%)	8420 (98.0%)	8469 (98.6%)	8497 (98.9%)	8513 (99.1%)
	Accuracy	SMILES	3331 (38.8%)	4147 (48.3%)	4479 (52.2%)	4681 (54.5%)	4812 (56.0%)
		SELFIES	1170 (13.6%)	1548 (18.0%)	1732 (20.2%)	1859 (21.6%)	1956 (22.8%)
		SAFE	1609 (18.7%)	2095 (24.4%)	2343 (27.3%)	2495 (29.1%)	2575 (30.0%)
		t-SMILES	80 (0.9%)	126 (1.5%)	162 (1.9%)	177 (2.1%)	193 (2.2%)
		fragSMILES	**3805 (44.3%)**	**4349 (50.6%)**	**4656 (54.2%)**	**4830 (56.2%)**	**4962 (57.8%)**
Retro-synthesis (chiral)	Validity	SMILES	3427 (39.9%)	4580 (53.3%)	5559 (64.7%)	6261 (72.9%)	6770 (78.8%)
		SELFIES	**6886 (80.2%)**	**7645 (89.0%)**	**7990 (93.0%)**	**8144 (94.8%)**	**8230 (95.8%)**
		SAFE	3823 (44.5%)	4793 (55.8%)	5563 (64.8%)	6082 (70.8%)	6524 (76.0%)
		t-SMILES	6167 (71.8%)	6817 (79.4%)	7316 (85.2%)	7597 (88.5%)	7801 (90.8%)
		fragSMILES	4485 (52.2%)	5678 (66.1%)	6452 (75.1%)	6958 (81.0%)	7318 (85.2%)
	Accuracy	SMILES	**656 (7.6%)**	918 (10.7%)	1094 (12.7%)	1236 (14.4%)	1331 (15.5%)
		SELFIES	0 (0.0%)	0 (0.0%)	0 (0.0%)	0 (0.0%)	0 (0.0%)
		SAFE	635 (7.4%)	805 (9.4%)	924 (10.8%)	1048 (12.2%)	1125 (13.1%)
		t-SMILES	0 (0.0%)	0 (0.0%)	0 (0.0%)	0 (0.0%)	0 (0.0%)
		fragSMILES	620 (7.2%)	**918 (10.7%)**	**1127 (13.1%)**	**1296 (15.1%)**	**1467 (17.1%)**

Sup. Table 5 Analysis of prediction accuracy on two distinct chiral subsets of the test set, selected based on specific chirality token patterns in fragSMILES. Accuracy values are referred to the Top-1 forward prediction task.

<table>
<tr><th>Set description</th><th>Set size</th><th>Notation</th><th>Accuracy</th></tr>
<tr><td rowspan="5">Chemical reactions where the pathway favors the formation of a specific stereoisomer over others</td><td rowspan="5">2059</td><td>SMILES</td><td>183 (8.9%)</td></tr>
<tr><td>SAFE</td><td>79 (3.8%)</td></tr>
<tr><td>SELFIES</td><td>55 (2.7%)</td></tr>
<tr><td>t-SMILES</td><td>2 (0.1%)</td></tr>
<tr><td>fragSMILES</td><td>132 (6.4%)</td></tr>
<tr><td rowspan="5">Reactants and chemical product preserve same set of tokens for chirality reporting (e.g. C|S, <4S>, etc)</td><td rowspan="5">6148</td><td>SMILES</td><td>2942 (47.9%)</td></tr>
<tr><td>SAFE</td><td>1422 (23.1%)</td></tr>
<tr><td>SELFIES</td><td>1041 (16.9%)</td></tr>
<tr><td>t-SMILES</td><td>69 (1.1%)</td></tr>
<tr><td>fragSMILES</td><td>3669 (59.7%)</td></tr>
<tr><td rowspan="5">Reactants or chemical product involve a ring represented with chirality as suffix (e.g., C1CC2COC(C1)O2|2R5R)</td><td rowspan="5">1350</td><td>SMILES</td><td>361 (26.7%)</td></tr>
<tr><td>SAFE</td><td>173 (12.8%)</td></tr>
<tr><td>SELFIES</td><td>84 (6.2%)</td></tr>
<tr><td>t-SMILES</td><td>24 (1.8%)</td></tr>
<tr><td>fragSMILES</td><td>403 (29.9%)</td></tr>
</table>

References

[1] D. Lowe, *Chemical reactions from US patents (1976-Sep2016)*, 2017, https://figshare.com/articles/dataset/Chemical_reactions_from_US_patents_1976-Sep2016_/5104873.

[2] A. Vaswani, arXiv, 2017.
